# Development and Initial Evaluation of an Internet-Based Support System for Face-to-Face Cognitive Behavior Therapy: A Proof of Concept Study

**DOI:** 10.2196/jmir.3031

**Published:** 2013-12-10

**Authors:** Kristoffer NT Månsson, Erica Skagius Ruiz, Elisabet Gervind, Mats Dahlin, Gerhard Andersson

**Affiliations:** ^1^PsychologyDepartment of Behavioral Sciences and LearningLinköping UniversityLinköpingSweden; ^2^Psykologpartners W&W ABLinköpingSweden; ^3^Psychiatry SectionDepartment of Clinical NeuroscienceKarolinska InstitutetStockholmSweden

**Keywords:** cognitive behavior therapy, Internet, anxiety, depression, Apple iPad

## Abstract

**Background:**

Evidence-based psychological treatments, such as cognitive behavior therapy (CBT), have been found to be effective in treating several anxiety and mood disorders. Nevertheless, issues regarding adherence are common, such as poor patient compliance on homework assignments and therapists’ drifting from strictly evidence-based CBT. The development of Internet-delivered CBT (ICBT) has been intensive in the past decade and results show that guided ICBT can be as effective as face-to-face CBT but also indicate a need to integrate the two forms of CBT delivery.

**Objective:**

In this study, we developed and tested a new treatment format in which ICBT and face-to-face therapy were blended. We designed a support system accessible via the Internet (using a computer or an Apple iPad) for patients and therapists delivering CBT face-to-face. The support system included basic CBT components and a library of interventions gathered from existing ICBT manuals.

**Methods:**

The study involved 15 patients with mild to moderate anxiety or depression (or both). Eight therapists conducted the treatments. All participants were interviewed after the nine-week intervention. Further, patients provided self-reports on clinical measures pre- and post-trial, as well as at a 12-month follow-up.

**Results:**

A reduction was found in symptom scores across all measures. The reliable change index ranged from 60% to 87% for depression and anxiety. Large effect sizes (Cohen’s *d*) ranging from 1.62 (CI 95% 0.59-2.66) to 2.43 (CI 95% 1.12-3.74) were found. There were no missing data and no treatment dropouts. In addition, the results had been maintained at the 12-month follow-up. Qualitative interviews revealed that the users perceived the support system as beneficial.

**Conclusions:**

The results suggest that modern information technology can effectively blend with face-to-face treatments and be used to facilitate communication and structure in therapy, thus reducing therapist drift.

##  Introduction

The lifetime prevalence of mood and anxiety disorders is high, ranging from 20.8% to 28.8% [1], and these disorders are associated with significantly reduced quality of life in sufferers [2]. According to the World Health Organization (WHO), by the year 2030 depression is expected to be the largest contributor to disease burden [3]. Thus, there is a need for cost-effective and accessible treatments. Evidence-based treatments exist and both pharmacotherapy and psychological treatments (mainly cognitive behavior therapy; CBT) have established evidence bases [4]. However, access to CBT, often the preferred treatment option [5], is limited; thus, a gap remains between demand for treatment and actual provision [6]. Moreover, evidence-based psychological treatments do not help all patients and issues regarding adherence to therapy are common [7]. Dropout from treatment, poor engagement, and incomplete adherence to homework assignments are all issues that can reduce the treatment’s effectiveness [8]. Further, therapist drift from evidence-based CBT (toward supportive therapy) has been highlighted as a potential obstacle to effective CBT [9]. Hence, ways to optimize the delivery of CBT, improve response rates, and increase the treatment’s availability represent important goals for the field.

Research on Internet-delivered cognitive behavior therapy (ICBT) has been intensive in the past decade and studies indicate that clinician-guided ICBT can be as effective as face-to-face CBT for some diagnoses [10,11]. ICBT has been developed for several mood and anxiety disorders [12,13] and has also been implemented in regular clinical psychiatric settings [14]. Several clear advantages can be noted when comparing ICBT with traditional, face-to-face CBT [15]. Obviously, less time is needed when the treatment is delivered via the Internet [16]; hence, more patients can be reached at a lower cost. In addition, the rationale and content of ICBT adhere to treatment manuals closely and, even though there is a role for the therapist in guided ICBT [17], this treatment format is probably less susceptible to therapist drift. On the other hand, some problems with ICBT should be noted. Therapies with minimal support may lead to increased dropout rates (particularly in unguided ICBT) [18], and some patients probably need more support than ICBT provides in order to adhere to the therapy [19]. Face-to-face meetings with patients have the advantage of providing additional clinically relevant information that can be used for diagnostic purposes and individual case management. Moreover, when patients need support in order to understand therapeutic techniques, face-to-face contact is beneficial (as a treatment technique can be illustrated directly during the session).

Based on our research experience examining both treatment-delivery methods—face-to-face and via the Internet—we sought to merge the two to create a blended treatment format. Previous studies have combined computerized manuals with face-to-face treatment [20,21], used technological adjuncts in therapy [22], or used the Internet after treatment completion to help prevent relapse [23,24]. To our knowledge, this is the first study to merge ICBT and face-to-face CBT in a system used by both the therapist and the patient. Accordingly, we constructed a Web platform accessible via personal computer and Apple iPad and blended ICBT with traditional face-to-face CBT. This case series pilot study aimed to explore clinical outcomes on measures of anxiety, depression, and quality of life when Internet-delivered support was provided to patients and their therapists. In addition, we also examined user experiences and the ways that the support system was used and perceived. We hypothesized that the support system would contribute positively to adherence and that outcomes would align with those achieved in face-to-face CBT. This study was designed as a proof-of-concept study, generating ideas for further research.

## Methods

### Procedure

The local ethics committee approved the study (ID: 2011/456-31). Fifteen participants fulfilling all inclusion criteria were notified via email of their acceptance and gave written informed consent prior to treatment. The treatment duration was between eight and nine weeks. In combination with the last session, a qualitative interview was conducted with the patient. On this occasion, each patient was also directed to a website where he or she was to fill out self-report questionnaires representing the clinical outcome measures. At post-treatment, seven therapists participated in a focus group discussing user experiences. One individual interview was conducted with another therapist. In addition, long-term follow-up clinical data were obtained at a 12-month follow-up with each patient.

### The Support System

The platform (support system) utilized in the study was conceived and designed by the first and the last authors, KM and GA. A programmer wrote the code and used the open-source code language PHP and MySQL to construct the platform, which was accessible via personal computer or Apple iPad through an encrypted secure sockets layer (SSL) connection to the Internet. The support system was not developed as a mobile phone application. Users were assigned personal log-in IDs via email and an additional temporary password was sent via SMS (short message service) at each attempt to log on (this feature was implemented in order to increase the level of security). The platform was to be used at home and in the clinical setting where treatment was provided. The platform was set up so that the user would receive part of the information that would have been provided in a face-to-face treatment session—in other words, there were no computerized treatment components and the main part of the system consisted of material that in a face-to-face session would have been presented on printed paper or verbally (eg, goal setting). The therapists and patients did not access the same content in the platform; therapists controlled which support resources and information patients had access to. The platform contained some basic components of CBT, such as scheduling visits, keeping an agenda, and setting goals. The platform also included a library with both text and media resources used in psychoeducation and as homework assignments. These resources were compiled primarily from prior studies on ICBT for anxiety [25] and depression [26]; they were presented not as discrete treatments but rather as part of the face-to-face treatment (ie, as handouts). Use of text material in face-to-face treatment has been found to be common among CBT practitioners [27] and we regarded this procedure as aligning with standard CBT. The digital library contained supplemental information on CBT, such as behavioral activation, activity scheduling, exposure therapy, common cognitive biases, and maintenance via safety behaviors. We also provided some audio files, such as relaxation instructions. In addition, the platform included common questionnaires and forms used in homework assignments, such as guides to creating a fear hierarchy and keeping daily thought records and sleep diaries.

**Figure 1:**
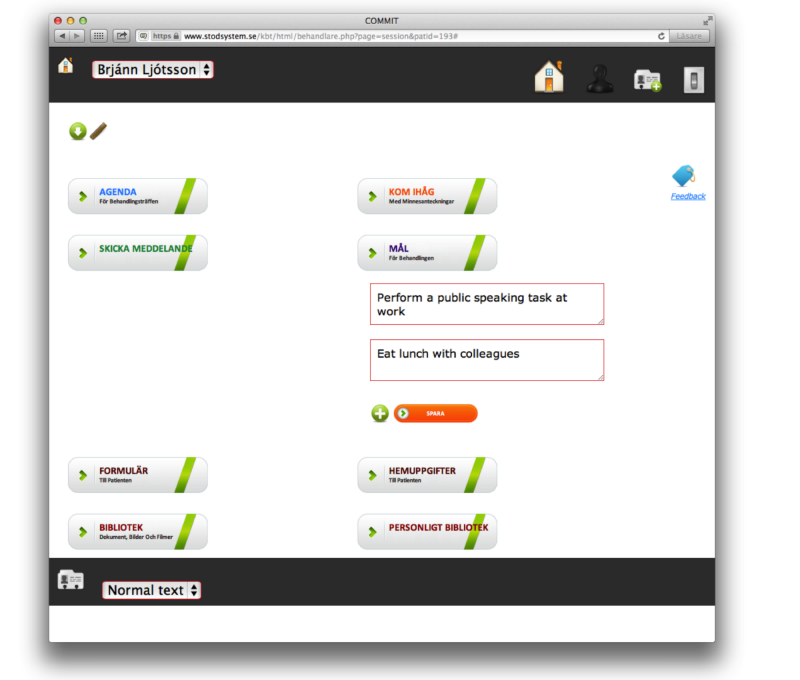
Screenshot of the Internet-based support system.

In addition, the users could communicate via an internal message system and the therapists could communicate via mobile phone SMS. However, communication between users was essentially made within the support system. Users could also create memos about topics they wished to remember or discuss in therapy and they were able to upload and share personal files. The Web platform was built to give support to both therapists and patients in the delivery of face-to-face CBT. For further details, see Figure 1 and Multimedia Appendix 1.

### Recruitment

The study involved 23 participants in total: 15 patients and 8 therapists. Patients were recruited mainly via local media advertising and information distributed on a university campus. Participants declared their interest via a research project website and provided informed consent. Therapists were recruited separately via email.

### Participants

The patients’ mean age was 43 years old (SD 15) and ages ranged from 22 to 70 years. A majority of participating patients (60%, 9/15) were currently university students or previously had been. Table 1 presents the demographic characteristics and Table 2 the distribution of psychiatric diagnoses. Of the eight therapists who were involved in the study, seven had recently received basic psychotherapy training as part of the final year of their five-year clinical psychology program. One therapist was in his first year of clinical practice as a psychologist. The mean time of experience in CBT under supervision was 14 months (range 4-26 months). All the therapists volunteered and did not receive any compensation.

A total of 26 patients were interviewed via telephone using the structured diagnostic psychiatric interview for Diagnostic and Statistical Manual of Mental Disorders (DSM-IV) and International Classification of Diseases (ICD-10): Mini-International Neuropsychiatric Interview (MINI [28]). Two psychology students in their final term conducted the clinical interviews. Both students had experience with diagnostic interviews and basic training in CBT. After the interviews, participants were instructed to fill out self-reports measuring anxiety, depression, quality of life, and alcohol consumption via a secured and encrypted website.

The following participant inclusion criteria were applied in the study: (1) over 18 years of age, (2) no history of ongoing suicidal attempts or current tendency toward self-harm, (3) no current alcohol or drug addiction, (4) no current contact with a physician administering pharmacotherapy that required monitoring, (5) if the patient was on pharmacotherapy, the dose had been stable for at least one month before the study began and the participant agreed to keep the dosage unchanged during the study or inform the therapist if a change was needed, (6) not receiving other concurrent psychological treatment, (7) not diagnosed with severe major depression disorder according to MADRS-S (Montgomery Åsberg Depression Rating Scale − self-report) (cut-off at >30 points), (8) reported <4 on MADRS-S item 9 (about suicidal ideations), (9) not suffering from a severe psychiatric condition that could interfere with the treatment (eg, bipolar disorder or schizophrenia, as reported in the clinical interview), and (10) access to the Internet via a computer. A senior researcher (licensed psychologist and psychotherapist) reviewed participants to determine their eligibility for inclusion.

**Table 1:** Demographic characteristics (N=15).

Characteristic		Mean (SD) or n (%)
Age, mean (SD)		43.0 (15)
Gender, female, n (%)		10 (67)
**Educational level, n (%)**		
	Completed university	7 (47)
	Completed vocational training	4 (27)
	Current university	2 (13)
	Completed high school	2 (13)
**Employment status, n (%)**		
	Working	9 (60)
	Student	4 (27)
	Retired	2 (13)
**Current pharmacotherapy, n (%)**		
	SNRI^a^	4 (27)
	SSRI^b^	2 (13)
	Sedatives	2 (13)
**Previous experience of therapy, n (%)**		
	CBT^c^	4 (27)
	Unspecified therapy	3 (20)
	Single CBT interventions	2 (13)
	Counseling	2 (13)
**Computer experience, n (%)**		
	Good	13 (87)
	Limited	2 (13)

^a^SNRI: serotonin and norepinephrine reuptake inhibitors

^b^SSRI: selective serotonin reuptake inhibitors

^c^CBT: cognitive behavior therapy

**Table 2:** The distribution of diagnoses according to the MINI psychiatric interviews (N=15).

Diagnosis	Frequency, n (%)
Major depressive episode	6 (40)
Social anxiety disorder	5 (33)
Generalized anxiety disorder	4 (27)
Agoraphobia	3 (20)
Panic disorder	2 (13)
Comorbidity (participants fulfilling two or more diagnostic criteria)	5 (33)

### Cognitive Behavior Therapy

The individualized CBT was delivered during a period of eight to nine weeks. The treatments were based on generic CBT components, such as case conceptualization, behavior analysis, agenda setting, goal setting, psychoeducation, and homework assignments. Therapists received clinical group supervision from a licensed clinical psychologist during four scheduled appointments during the trial. In addition, on four occasions the therapists and the research project manager met and discussed experiences and feedback regarding technical issues. Each therapist and patient pair individually decided how to use the platform during the treatment—for instance, during therapy sessions and in additional contact between sessions.

### Clinical Measures

We used the Beck Anxiety Inventory (BAI) [29] and the Generalized Anxiety Disorder Screener-7 (GAD-7) [30] to measure anxiety. The Montgomery Åsberg Depression Rating Scale-self-rating version (MADRS-S) [31] and the Patient Health Questionnaire-9 (PHQ-9) [32] were used to measure symptoms of depression. In addition, the Quality of Life Inventory (QOLI) [33] was administered. All clinical measures have established good psychometric properties when administered via the Internet [34,35]. We also used the Alcohol Use Disorder Identifications Test (AUDIT) [36] for screening purposes but not as an outcome measure.

### Statistical Analysis

Data were analyzed with SPSS version 21. All the statistical analyses were intent-to-treat and statistical significance was set at *P*<.05. Differences between pre- and post-treatment measures and pre-treatment and long-term follow-up measures of depression, anxiety, and quality of life were examined using paired sample *t* tests. Within-group effect sizes were calculated based on the pooled standard deviation, expressed as Cohen’s *d*.

We used a reliable change index (RC) in accordance with Jacobson and Truax [37] to determine the proportion of participants who showed treatment improvements from pre- to post-treatment and from pre-treatment to long-term follow-up. As suggested by Lambert and Ogles [38], we used internal consistency as a measure of reliability. The following values were used in the calculation of RC: Cronbach alpha=.92 for BAI, GAD-7, and PHQ-9 [29,30,39]; Cronbach alpha=.87 for MADRS-S [40]; and Cronbach alpha=.83 for QOLI [33].

### Qualitative Analysis

This part of the study was based on content analysis in order to analyze the data [41]. Individual interviews and the focus group were recorded and transcribed. All interviews were recorded using a portable digital recorder. The purpose of the interviews was to identify user experiences of the platform used in the treatment. Meaningful units from the text based on the research question were coded and organized into categories. Sentences that reflected opinions, such as adjectives and words that expressed an emotive experience were selected. Meaningful units and user experiences were sorted as positive, negative, and neutral. Topics that were mentioned frequently and explicitly became the basis for the categorization. Each patient interview (n*=*15) was merged and overarching themes were created (see Table 3). Overarching themes were formulated in an ongoing discussion between two researchers, facilitating the development of supplementary views and critical perspectives. The same procedure of qualitative analysis was applied in analyzing both the focus group discussions and the individual therapists’ comments.

**Table 3:** Overarching themes identified in the qualitative interviews with patients and therapists.

Connotation		Feedback
**Patient interviews**		
	−^a^	Computer skills influenced the work with the support system
	+^b^	Facilitating treatment outside the therapy room
	+	Memory support and learning
	+	Positive experiences of the treatment
	+	Positive implications for homework assignments
	+	Potential to gain an overview of the treatment process
	+	Promoted a sense of autonomy and responsibility
	+	Supported maintenance after therapy
	+	The library—an individualized supplement
	+	The use of the support system during and between sessions
	+/−^c^	The iPad was not seen as an obstacle
	+/−	Working with digital material—helpful or unnecessary
**Therapist interviews**		
	−	For patients with less computer experience, ICBT hampered the work
	+	Increased therapist skills by providing overview of the therapy process
	+	Positive experience of communication between sessions
	+	Positive experiences using the support system
	+	The library—an important support
	+	The support system promoted additional structure in the therapy
	+	The use of the support system during and between sessions
	+/−	Heterogeneity in amount of time engaging in treatment
	+/−	Pros and cons of the support system as a substitute to the face-to-face sessions
	+/−	Pros and cons of using the support system in face-to-face sessions
	+/−	The support system affected the therapists’ workload

^a^ −, negative feedback

^b^ +, positive feedback

^c^ +/−, positive and negative feedback

##  Results

### Clinical Outcome Measures

**Figure 2:**
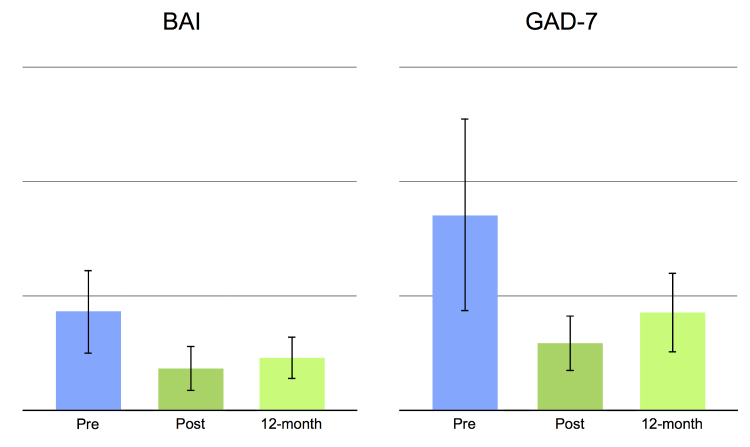
Histograms showing clinical measures of anxiety. Mean values at pre-treatment, post-treatment, and at 12-month follow-up. Error bars represent the standard deviation.

**Figure 3:**
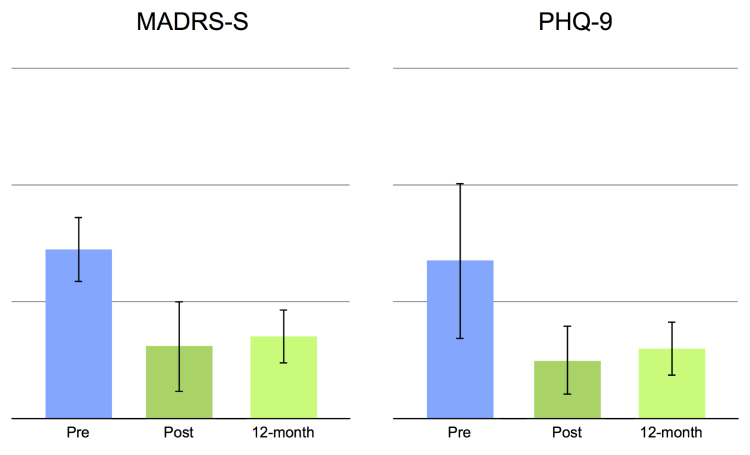
Histograms showing clinical measures of depression. Mean values at pre-treatment, post-treatment, and at 12-month follow-up. Error bars represent the standard deviation.

**Figure 4:**
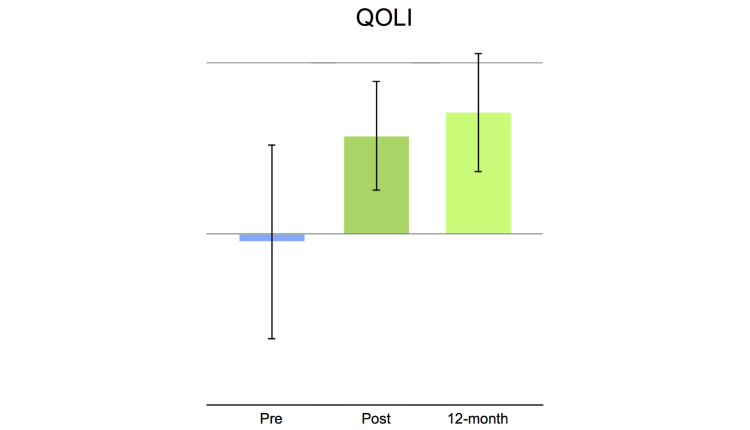
Histogram showing clinical measures on quality of life. Mean values at pre-treatment, post-treatment, and at 12-month follow-up. Error bars represent the standard deviation.

This study had no missing data and no treatment dropouts. Statistically significant main effects were obtained for all clinical outcome measures, pre- to post-treatment, *t*_14_=4.25 to 7.25, all *P*’s<.001, and pre- to long-term follow-up, *t*_14_=3.53 to 6.20, all *P*’s<.05 (see also Table 4 and Figures 2-4). Moreover, comparing mean values post-treatment to long-term follow-up were nonsignificant: *t*_14_=0.73 to 1.55, all *P*’s>.142. Large within-group effect sizes (Cohen’s *d*) were observed on all clinical outcome measures, with pre- to post-effect sizes ranging from 1.26-2.43 and long-term follow-up between 1.08-1.94 (Table 4). Reliable change as defined by Jacobson and Truax [34] was observed in a majority of the patients, ranging from 60% to 87% on depression and anxiety measures and from 53% to 80% at long-term follow-up (Table 4). Reliable change on quality of life was 40% at post-treatment and 60% at 12-month follow-up.

**Table 4:** Mean, SD, effect size, and reliable change index at pre-treatment, post-treatment, and at long-term follow-up to treatment.

		Pre-treatment (n=15)	Post-treatment (n=15)	Long-term follow-up^a^ (n=15)
**BAI** ^b^				
	Mean (SD)	18.07 (7.7)	7.67 (4.2)	9.60 (7.8)
	Effect size (CI 95%)	—	1.67 (0.6-2.74)	1.09 (0.39-1.79)
	Reliable change, n (%)	—	11 (73)	9 (60)
**GAD-7** ^c^				
	Mean (SD)	11.93 (5.9)	4.07 (1.7)	6.00 (4.9)
	Effect size (CI 95%)	—	1.80 (0.64-2.97)	1.08 (0.32-1.84)
	Reliable change, n (%)	—	9 (60)	8 (53)
**MADRS-S** ^c^				
	Mean (SD)	21.20 (4.1)	9.07 (5.7)	10.27 (6.7)
	Effect size (CI 95%)	—	2.43 (1.12-3.74)	1.94 (0.9-2.98)
	Reliable change, n (%)	—	13 (87)	12 (80)
**PHQ-9** ^d^				
	Mean (SD)	12.13 (6.0)	4.46 (2.7)	5.33 (4.2)
	Effect size (CI 95%)	—	1.62 (0.59-2.66)	1.31 (0.32-2.31)
	Reliable change, n (%)	—	11 (73)	10 (67)
**QOLI** ^e^				
	Mean (SD)	−0.13 (1.6)	1.60 (0.9)	1.98 (1.6)
	Effect size (CI 95%)	—	1.26 (0.49-2.02)	1.29 (0.41-2.18)
	Reliable change, n (%)	—	6 (40)	9 (60)

^a^Effect size and the reliable change index were calculated based on pre-treatment data versus long-term follow-up data.

^b^BAI: Beck Anxiety Inventory.

^c^GAD-7: Generalized Anxiety Disorder questionnaire (7-item version).

^d^MADRS-S: Montgomery Åsberg Depression Rating Scale (self-report version).

^e^PHQ-9: Patient Health Questionnaire (9-item version).

^f^QOLI: Quality of Life Inventory.

### Patients’ User Experiences

The patients reported that they had used the various featured components of the support system. A majority said that the platform was easy to use; however, two patients with less computer experience had used the platform only sporadically. No patient regarded the iPad as a disturbing feature of the therapy. Thirteen patients reported that they had carried out all or almost all their homework assignments; two patients reported that they had carried out more than half their assignments. The availability of assignments on the Internet was described as easy, convenient, and modern, and patients indicated that it prevented avoidance of feared situations (eg, in exposure therapy tasks). About half the patients appreciated having online feedback between face-to-face sessions. One patient had more frequent contact with the therapist during a period of challenging homework assignments. Some also found the process of writing about their problems to be helpful. Most of the patients expressed largely positive opinions about sharing information and homework over the Internet. One-third of the patients also reported that they spent additional time reading other information on the Internet related to the therapy. The content in the library was perceived as a supplement to the treatment, promoting theoretical understanding and enhanced knowledge. Several patients highlighted the use of the platform as a memory aid. One patient described the educational advantages of obtaining information through several media, such as speech, writing, and images. Another reported advantage of the Internet-based platform was the patients’ ability to return to previous assignments and reflect on their experience of change, which was perceived as motivating. A few patients indicated that the treatment contributed to increased participation and responsibility for their treatment and that both the therapist and patient collaboratively created the homework assignments with the help of the support system.

The treatment was said to facilitate the generalization of treatment to the patients’ everyday lives. Several patients perceived the treatment duration as longer and more extensive than they had expected a nine-week intervention to be. About one-third of the patients expressed ambivalence when it came to continued work on their own. One patient also suggested developing this concept so that it could be used after treatment in order to maintain treatment gains.

### Therapists’ User Experiences

The self-reported estimation of time spent with each therapy and week ranged between 15 and 180 minutes (mean 77, SD 57). Seven therapists (87.5%, 7/8) estimated that they spent less or as much time in these treatments, compared to treatments without the platform. The therapists accessed the platform on average seven times per patient per week. However, this number also includes the pre-treatment phase, during which the therapists were introduced to the support system. The therapists confirmed that they had used all the components of the platform. Internal messages and correspondence via SMS were frequently used. Messages contained clarifications and reformulations of homework assignments, providing support, encouragement, and answers to patients’ questions. Correspondence via the platform was also used to remind patients about assignments. This resulted in the use of the platform when therapy sessions were cancelled. The therapists were able to help the patients relatively rapidly instead of waiting until the next appointment. This may have contributed to the therapy’s continuity, allowing therapists to prepare the face-to-face sessions based on the results of a patient’s weekly assignments. One therapist also highlighted that the platform made it possible to focus more on the relational aspects of treatment during sessions. A majority of the therapists highlighted these possibilities as major benefits. Using the platform also contributed to structure in therapy. The structure was obtained when both therapist and patient could agree on and set an agenda, goals, and the central focus of homework assignments. The library was considered a source of knowledge and therapy-skill enhancement; it supported therapists in choosing among interventions and was helpful when therapists presented the treatment rationale. However, this easy access also led therapists to rely more on the content of the library material and to seek fewer other sources for help and supervision. The iPad was used to take notes, to draw, and to take pictures and videos during therapy sessions that could later be shared via the platform. Using the iPad during face-to-face sessions could potentially be clumsy and problematic, creating a barrier between therapist and patient, in particular if the therapist’s technical experience was limited. But using the iPad to take pictures of drawings on the wallboard was highlighted as beneficial. The patients’ level of computer experience was mentioned as important by the therapists, even if technology was not seen as a problem for a particular patient.

##  Discussion

### Principal Findings

The aim of this study was to investigate the combination of face-to-face CBT and an Internet-based support system. Overall, the therapies led to improvements on clinical measures and the platform was perceived as helpful. Clinical outcome data showed significant main effects and reliable changes on all measures and these results had been maintained at 12-month follow-up. The findings on the whole align with previous findings regarding computerized support in combination with face-to-face CBT [42]. The patients’ user experiences show that the platform and the iPad were regarded as beneficial. Several patients stated that they gained more from the treatment than they had originally expected to during the nine-week period. However, this might also be explained by the therapists’ level of involvement and may not directly relate to the availability of the platform. Obviously, some therapists were involved in the research process, but all the therapists had limited clinical experience and therefore it is reasonable to assume that the support system benefitted the delivery of the therapy. Indeed, this is a research question on its own, given that online therapist education is only in its infancy [43,44] and that direct support for novice therapists via the Internet is even less well researched. We also hypothesize that this support system might be a useful instrument for supervising therapists. If psychotherapy supervision focuses on enhancing interpersonal therapist skills, the support system, which emphasizes the importance of formulation and homework assignments, can serve as a complement in supervision.

Therapists found several major benefits to delivering treatment in this format—for instance, improved between-session communication and ease of sharing treatment-related information. These inputs highlight some important implications and advantages over traditional face-to-face treatments. Replacing missed sessions with Internet-delivered interventions adds continuity and the treatment thus becomes less vulnerable to factors such as physical health and logistical problems, such as travel distance. Further, this may have implications for structuring treatment, for it demonstrates the importance of the therapists being keen to set the agenda and it shows that homework assignments must be formulated clearly to provide clear, educative information. This study had no missing data, nor any dropouts from treatment. Hence, it is plausible that communication via the Internet between therapy sessions mitigates dropout rates. Further, our results suggest that Internet-based treatment delivery might decrease tendencies toward therapist drift [9] and might increase adherence to CBT treatment manuals.

### Limitations

Among the limitations of the present study is that the sample size was low, thus reducing statistical power. We did not include a wait list or an active control group and therefore had no control for time and repeated testing, which limits the generalizability of the findings. Also, the study design does not allow us to explore different factors that explain the outcome of the study. It is possible that certain features in the system, ie, stand-alone SMS communication between therapy sessions, could achieve similar clinical outcome results.

It is noteworthy that two therapists in the study were also involved in the research process and evaluation of the system. Even if our procedure is more biased than if independent researchers had conducted the interviews, the qualitative method, content analysis, provided us with the possibility of reviewing the comments and views of the participants.

The therapists in this study had relatively little experience delivering CBT and the included patients were recruited via media advertisement. Therefore, this method might not be equally applicable in a representative clinical setting. Moreover, some possible obstacles to the support platform emerged in this study. Limited computer experience is likely to make this treatment delivery format less efficient in some situations; indeed, not all the patients found the platform very easy to use. However, only two participants reported having limited computer experience and we did not include any additional evaluative questions. This implies that future studies should address the issue of computer experience in greater detail.

### Conclusions

In sum, this study indicates that Internet-based support can be used without compromising the effects of CBT and that it in fact provides a vehicle for communication and for the structure of the therapy. This may be important for new therapists in training, but it is also potentially valuable to experienced therapists, who can have a tendency to drift away from the treatment manuals. Future studies should investigate the relative merits of adding a support system to face-to-face therapy and whether doing so facilitates retention and long-term learning. Studies are also needed to investigate the use of Internet-delivered support in more regular clinical settings with practicing CBT therapists.

## Multimedia Appendix 1

Screenshots from the therapist’s view of the platform. When the patient enters the support system, the view shows only information that is relevant to him or her. The therapist controls the visibility of this content.

## Abbreviations

AUDIT: Alcohol Use Disorder Identifications Test
BAI: Beck Anxiety Inventory
CBT: cognitive behavior therapy
DSM-IV: Diagnostic and Statistical Manual of Mental Disorders, 4th Edition
GAD-7: Generalized Anxiety Disorder−seven item version
ICBT: Internet-based cognitive behavior therapy
ICD-10: International Statistical Classification of Diseases and Related Health Problems, version 10
MADRS-S: Montgomery Åsberg Depression Rating Scale−self-report version
MySQL: My Structured Query Language
PHP: hypertext preprocessor
PHQ-9: Patient Health Questionnaire−nine-item version
QOLI: Quality of Life Inventory
RC: reliable change index
RCT: randomized controlled trial
SMS: short message service
SNRI: serotonin and norepinephrine reuptake inhibitors
SSL: secure sockets layer
SSRI: selective serotonin reuptake inhibitors
WHO: World Health Organization
