# Supplementary material for: Development and Initial Evaluation of an Internet-Based Support System for Face-to-Face Cognitive Behavior Therapy: A Proof of Concept Study
Source: J Med Internet Res. 2013 Dec 10;15(12):e280. doi: 10.2196/jmir.3031 (PMC3868964; doi:10.2196/jmir.3031)
Supplement: Supplementary file 1 [file jmir_v15i12e280_app1.pdf]

Multimedia Appendix 1

Kristoffer NT Månsson[1], MSc; Erica Skagius Ruiz[1], MSc; Elisabet Gervind[1], MSc; Mats Dahlin[2], MSc; Gerhard Andersson[1,3], PhD

Affiliations

[1] Department of Behavioural Sciences and Learning, Linköping University, Sweden

[2] Psykologpartners W&W AB, Linköping, Sweden

[3] Department of Clinical Neuroscience, Psychiatry Section, Karolinska Institutet, Stockholm, Sweden

The following slides show screenshots from the therapist's view of the platform. When the patient: in this example, a character named Brjánn Ljótsson enters the support system, the view is slightly different, given that each patient should see only information that is relevant to him or her. The therapist controls the visibility of this content.

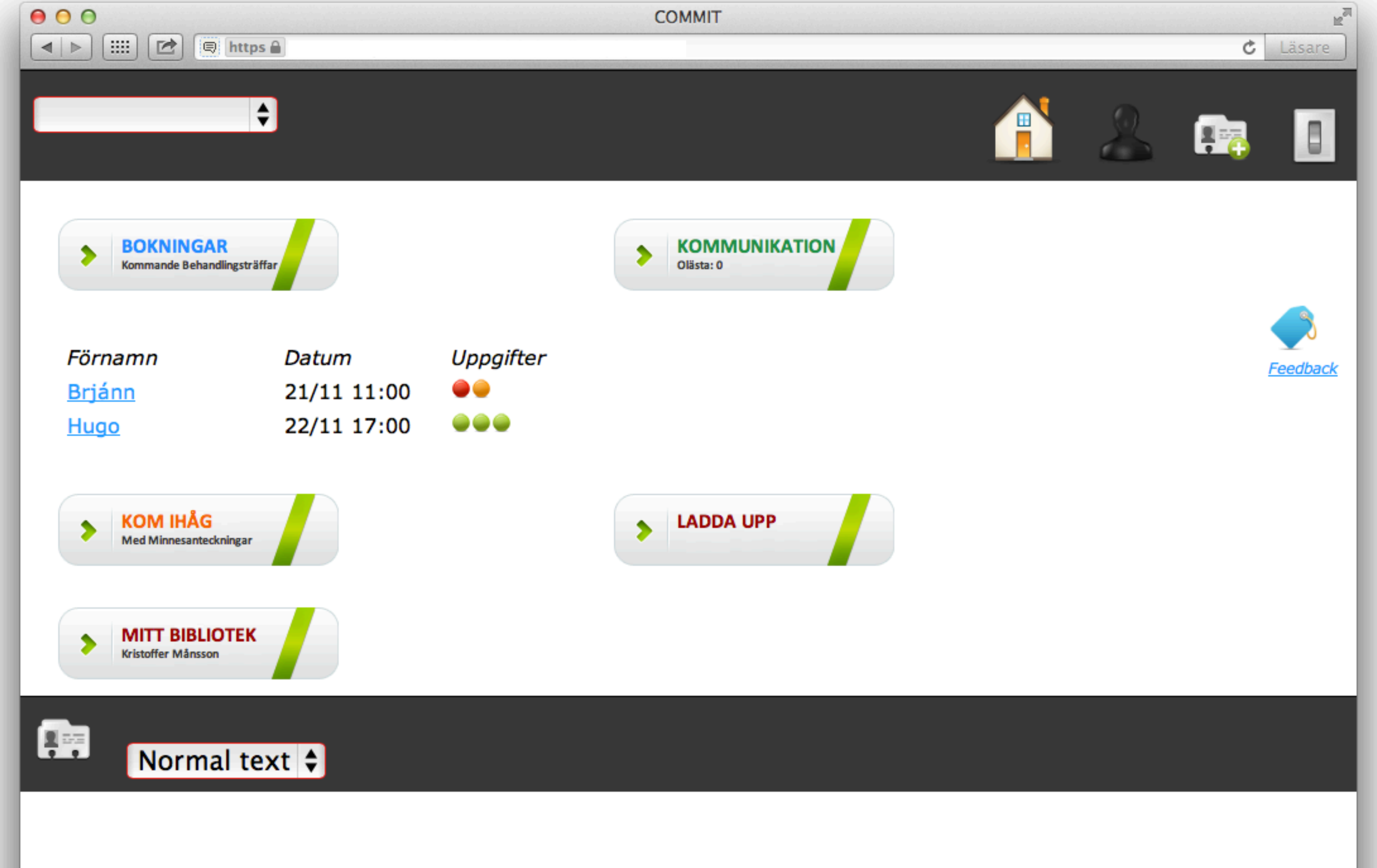
COMMIT
https
Läsare
BOKNINGAR
Kommande Behandlingsträffar
KOMMUNIKATION
Olästa: 0
Förnamn
Datum
Uppgifter
Brjánn
21/11 11:00
Hugo
22/11 17:00
Feedback
KOM IHÅG
Med Minnesanteckningar
LADDA UPP
MITT BIBLIOTEK
Kristoffer Månsson
Normal text

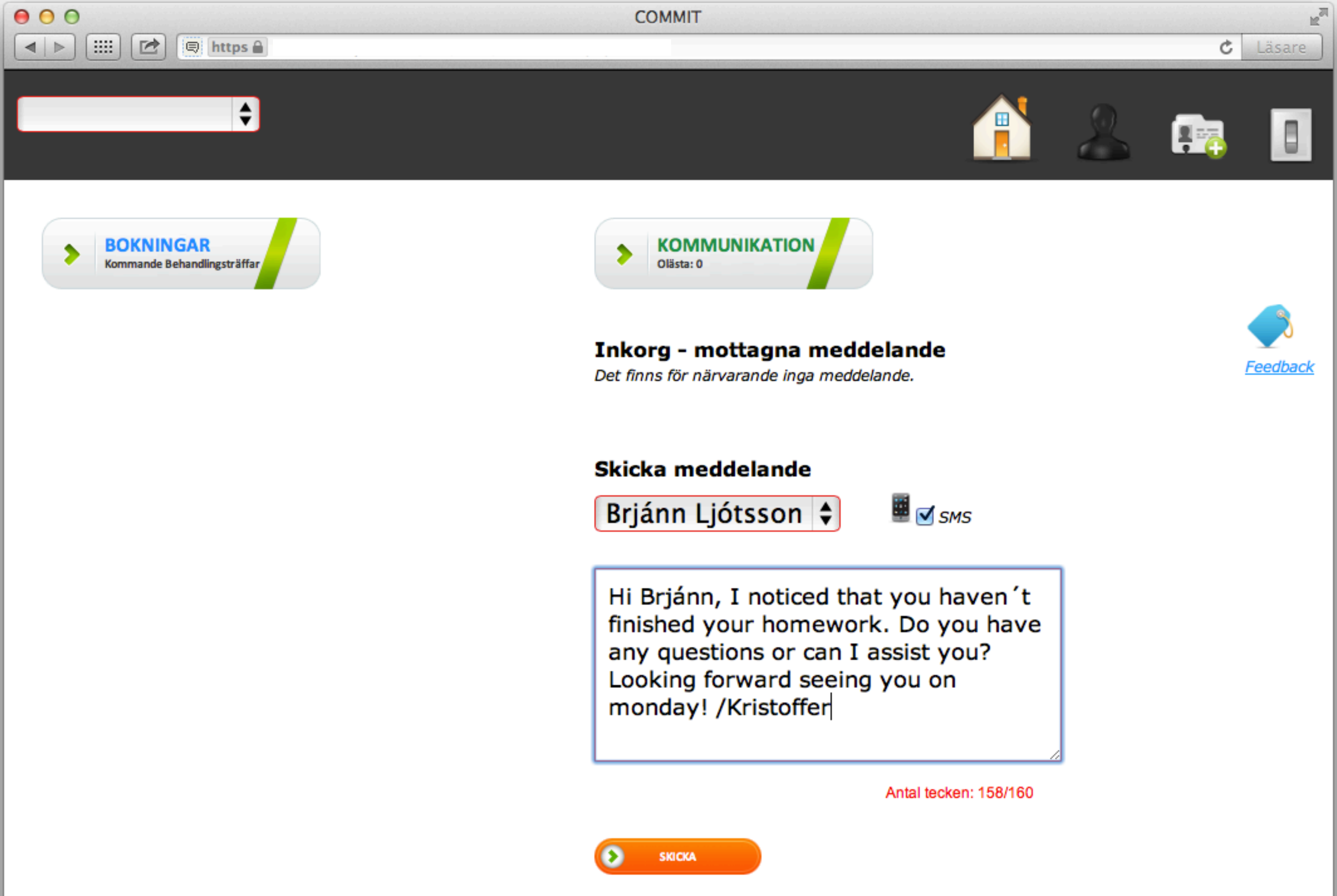
COMMIT
https
Läsare
BOKNINGAR
Kommande Behandlingsträffar
KOMMUNIKATION
Olästa: 0
Feedback
Inkorg - mottagna meddelande
Det finns för närvarande inga meddelande.
Skicka meddelande
Brjánn Ljótsson
SMS
Hi Brjánn, I noticed that you haven´t finished your homework. Do you have any questions or can I assist you? Looking forward seeing you on monday! /Kristoffer
Antal tecken: 158/160
SKICKA

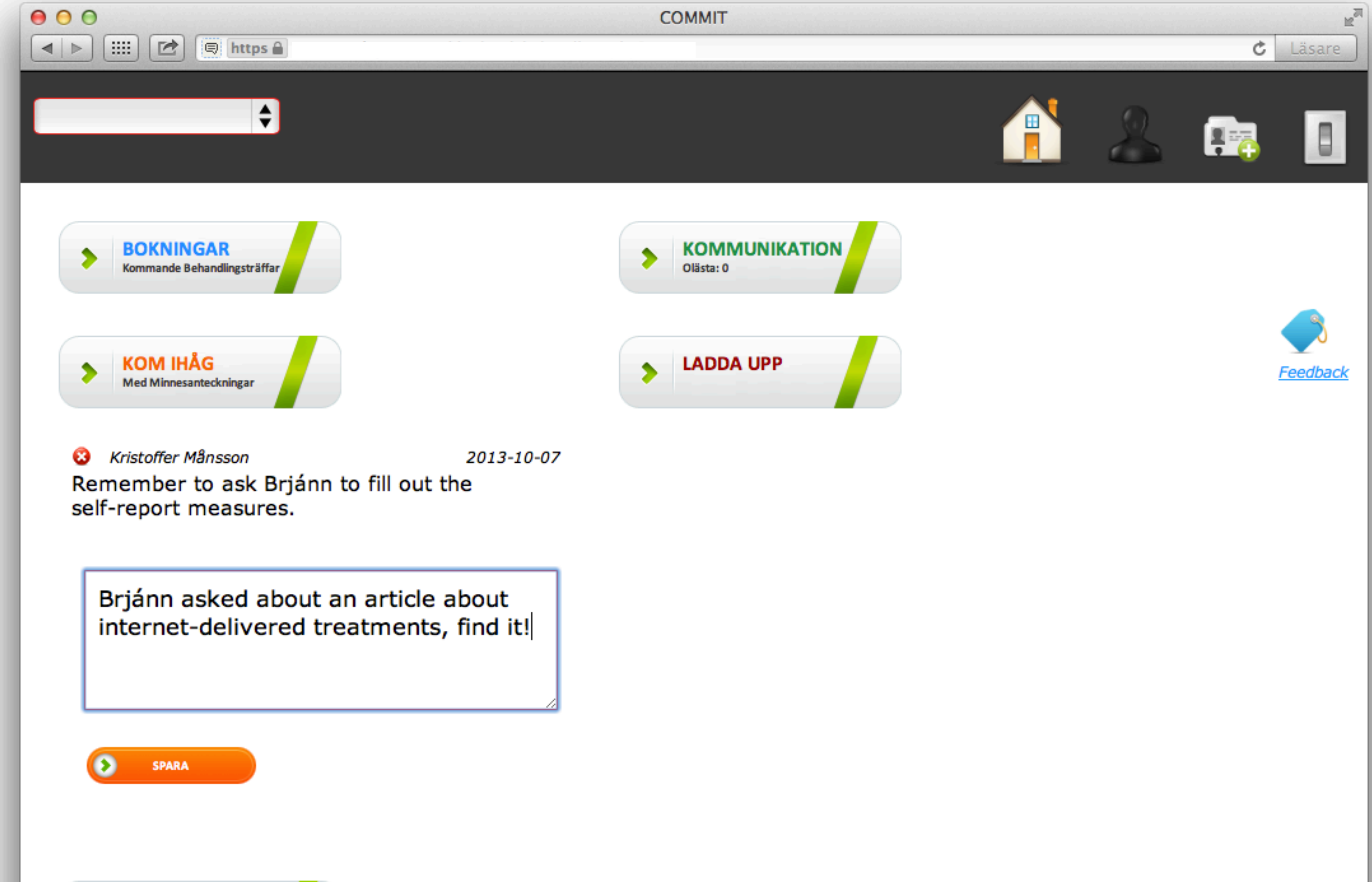
COMMIT
https
Läsare
BOKNINGAR
Kommande Behandlingsträffar
KOMMUNIKATION
Olästa: 0
KOM IHÅG
Med Minnesanteckningar
LADDA UPP
Feedback
Kristoffer Månsson
2013-10-07
Remember to ask Brjánn to fill out the self-report measures.
Brjánn asked about an article about internet-delivered treatments, find it!
SPARA

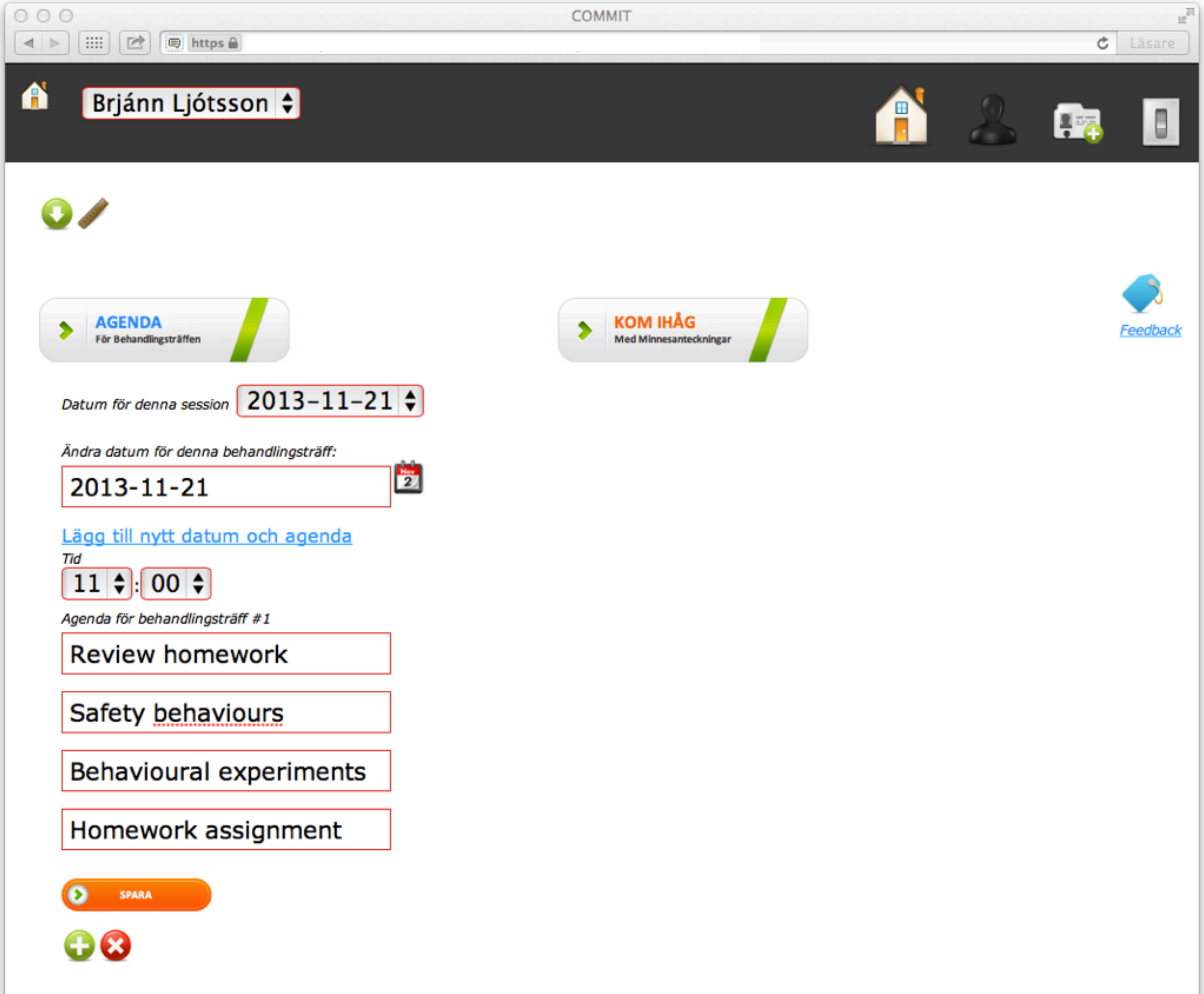
COMMIT
https
Läsare
Brjánn Ljótsson
AGENDA
För Behandlingsträffen
KOM IHÅG
Med Minnesanteckningar
Feedback
Datum för denna session
2013-11-21
Ändra datum för denna behandlingsträff:
2013-11-21
Lägg till nytt datum och agenda
Tid
11 : 00
Agenda för behandlingsträff #1
Review homework
Safety behaviours
Behavioural experiments
Homework assignment
SPARA

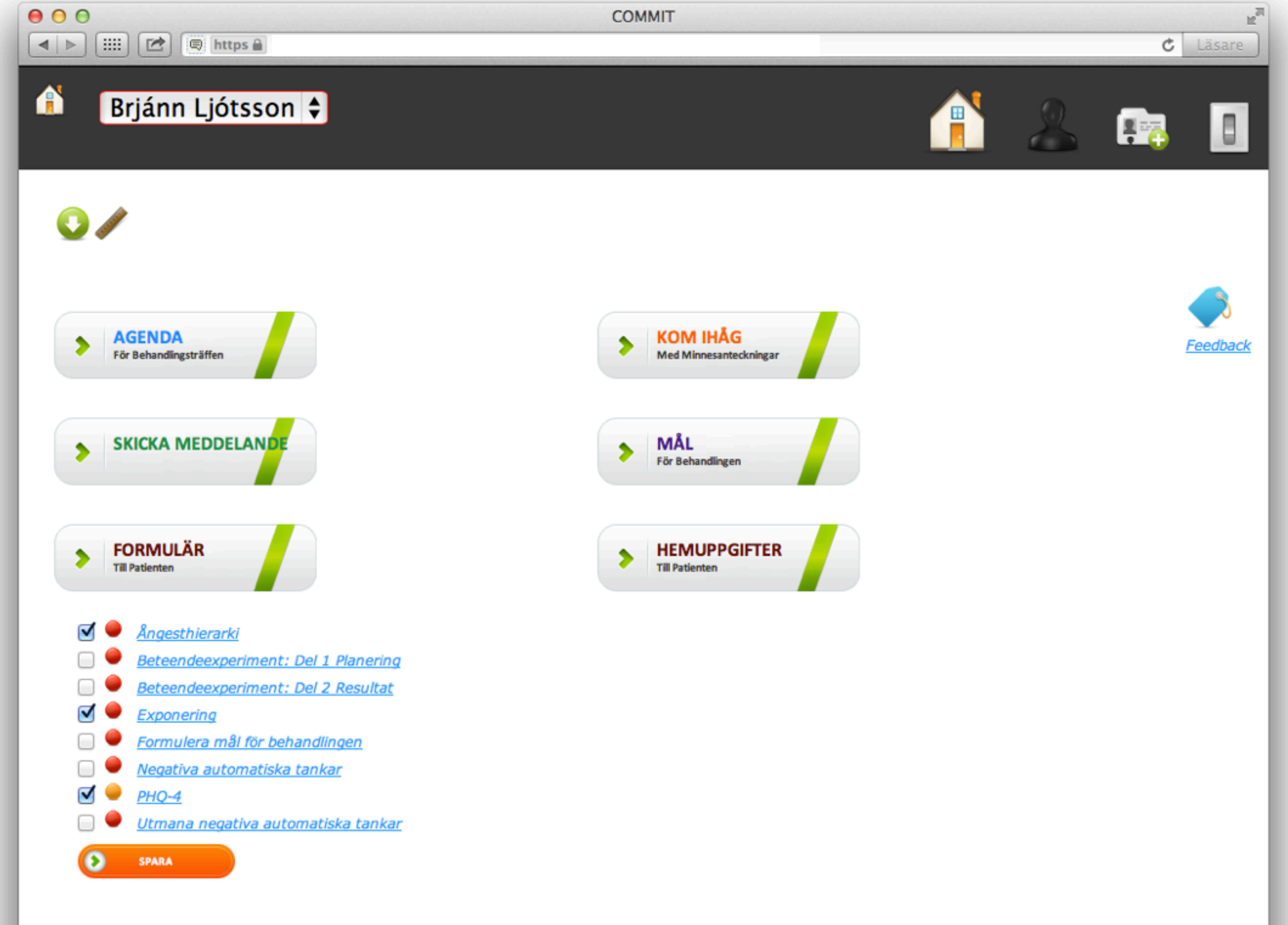
COMMIT
https
Läsare
Brjánn Ljótsson
AGENDA
För Behandlingsträffen
KOM IHÅG
Med Minnesanteckningar
Feedback
SKICKA MEDDELANDE
MÅL
För Behandlingen
FORMULÄR
Till Patienten
HEMUPPGIFTER
Till Patienten
Ångesthierarki
Beteendeexperiment: Del 1 Planering
Beteendeexperiment: Del 2 Resultat
Exponering
Formulera mål för behandlingen
Negativa automatiska tankar
PHQ-4
Utmana negativa automatiska tankar
SPARA

Feedback

AGENDA För Behandlingsträffen

KOM IHÅG Med Minnesanteckningar

SKICKA MEDDELANDE

MÅL För Behandlingen

FORMULÄR Till Patienten

HEMUPPGIFTER Till Patienten

BIBLIOTEK Dokument, Bilder Och Filmer

PERSONLIGT BIBLIOTEK

Aktiva filer

- [x] Demons on the boat ACT-metafor
- [x] A-aktiviteter psykoedukation.pdf
- [x] Kamp flykt ångest. Edukation.pdf

Övrigt

- [ ] Självförtroende.pdf
- [ ] Självförtroende.pdf

Ångest

Acceptans

- [ ] Acceptera i stunden en kortform.pdf
- [ ] Övning i acceptans av tankar och känslor.pdf

ACT

- [ ] ACT i ett nötskal.pdf
- [ ] Att mata en tiger.pdf

Avslappning

Swedish-English Glossary

- agenda: agenda
- bibliotek: library
- bokningar: scheduled visits
- datum: date
- formulär: forms
- förnamn: first name
- hemuppgifter: homework assignments
- kom ihåg: memos
- kommunikation: communication
- ladda upp: upload
- mål: goals
- uppgifter: assignments

Corresponding author: Kristoffer NT Månsson, kristoffer.nt.mansson@liu.se
